# A link between appendectomy and gastrointestinal cancers: a large-scale population-based cohort study in Korea

**DOI:** 10.1038/s41598-020-72770-5

**Published:** 2020-09-24

**Authors:** Youn Young Park, Kil-yong Lee, Seong Taek Oh, Sang Hyun Park, Kyung Do Han, Jaeim Lee

**Affiliations:** 1 Division of Coloproctology, Department of Surgery, College of Medicine, Uijeongbu St. Mary’s Hospital, The Catholic University of Korea, Cheonbo-ro 271, Uijeongbu-si, Gyeonggi-do 11765 Republic of Korea; 2 Department of Medical Statistics, College of Medicine, Catholic University of Korea, Seoul, Republic of Korea; 3 Department of Statistics and Actuarial Science, Soongsil University, Seoul, Republic of Korea

**Keywords:** Cancer, Gastroenterology, Risk factors

## Abstract

An association between appendectomy and subsequent gastrointestinal (GI) cancer development has been postulated, although the evidence is limited and inconsistent. To provide clarification, we investigated the link between appendectomy and GI cancers in a large nationwide appendectomy cohort. This cohort was derived from the claims database of the National Health Insurance Service in South Korea and comprised 158,101 patients who had undergone appendectomy between 2007 and 2014. A comparison cohort of 474,303 subjects without appendectomy was selected after 1:3 matching by age and sex. The incidence of GI cancers after appendectomy was observed, and risk factors for GI cancers were determined by using a multivariable-adjusted proportional hazards model. Appendectomy did not significantly increase the incidence of GI cancers in the overall population (1.529 and 1.557 per 1000 person-years in the non-appendectomy and appendectomy cohorts, respectively). However, appendectomy significantly increased the incidence of GI cancers in subgroups consisting of elderly (≥ 60 years) patients (adjusted HR, 1.102; 95% confidence interval, 1.011–1.201; *p* = 0.028) or women (adjusted HR, 1.180; 95% confidence interval, 1.066–1.306; *p* = 0.001).

## Introduction

Appendectomy is one of the most frequently performed surgical procedures. The belief that the appendix is a vestigial structure whose removal merely affects clinical outcome has been challenged over decades^1^. As a gut-associated lymphoid tissue, the appendix mediates host immune functions and hence might defend against enteric pathogens initiating malignant changes^1,2^.

The appendix also serves as a so-called “safe house” for biofilms, enabling preservation of the colonic microbiome^2–4^. Given the recently suggested link between the gut microbiome and gastrointestinal (GI) cancer, appendectomy might result in dysbiosis and consequent cancer development^5–14^. However, the association between appendectomy and GI cancer is controversial^1,15–18^, and epidemiologic reports using large databases to address this issue are limited in number^15,17^.

Therefore, we aimed to investigate the link between appendectomy and GI cancer development and to identify subgroups with a high risk of GI cancer by analysing a large-scale national appendectomy cohort in South Korea.

## Methods

### Study subjects and data collection

This study used the claims database of the National Health Insurance Service (NHIS), which was established for reimbursement of South Koreans covered by the NHIS or the medical aid program. The study protocol was approved by the official review committee of the National Health Insurance Corporation. Informed consent was waived by the Institutional Review Board of Uijeongbu St. Mary’s Hospital, Catholic University of Korea in South Korea (No. UC19ZESI0003).

**Figure 1:**
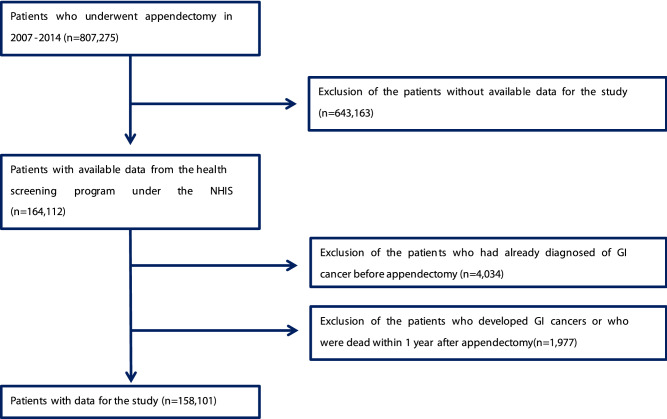
Flow chart for the selection of study cohort.

Using the NHIS claims database, we identified 807,275 patients who underwent appendectomy (NHIS procedure codes Q2861, Q2862, and Q2863) between 2007 and 2014 (Fig. 1). Among these patients, 164,112 had participated in the NHIS screening program. Demographic data retrieved from the program participants included age, sex, body mass index (BMI), smoking status, alcohol consumption, and history of hypertension, diabetes mellitus (DM), dyslipidaemia, and colorectal cancer. To avoid enrolling patients with pre-existing GI cancers, we excluded program participants with any GI cancers before appendectomy or who developed any GI cancers or died within 1 year after appendectomy. Finally, we identified an appendectomy cohort of 158,101 patients and a non-appendectomy cohort of 474,303 patients without appendectomy matched by age and sex (1:3).

The primary outcome was the number of newly developed GI cancers, which were defined in accordance with the International Classification of Diseases, 10th revision. The codes for oesophageal, gastric, small bowel, and colorectal cancer were C15, C16, C17, and C18–C20, respectively.

Obesity was defined as a BMI ≥ 25 kg/m^2^, and hypertension as a claim for antihypertensive agents owing to a diagnosis of I10–I15 or a systolic/diastolic blood pressure ≥ 140/90 mmHg. DM was defined as a claim for antidiabetic agents owing to a diagnosis of E10–14 or a fasting glucose level ≥ 7 mmol/L according to the health screening program database. Dyslipidaemia was defined as a claim for antihyperlipidemic agents owing to a diagnosis of E78 or a total cholesterol level ≥ 6.21 mmol/L according to the health screening program database^19^.

### Statistical analyses

Student's t-test and the chi-square test were used to analyse continuous and categorical variables, respectively. Hazard ratios (HRs) and 95% confidence intervals (CIs) for the risk of GI cancers in the appendectomy vs. non-appendectomy cohort were determined using Cox proportional hazards regression models. For estimation of adjusted HRs (aHRs), a model adjusted for age, sex, BMI, smoking status, alcohol consumption, hypertension, DM, dyslipidaemia, and appendectomy was used. Kaplan–Meier curves for incidence probability were constructed and were compared using the log–rank test. A *p* value < 0.05 was considered significant. Statistical analyses were performed using SAS version 9.4 software (SAS Institute Inc., Cary, NC, USA).

## Results

### Demographic characteristics of the study population

The mean age of the patients in our study was 44.06 ± 14.09 years, and the median follow-up duration was 5.6 (3.78–7.59) years. The demographic characteristics of the appendectomy and non-appendectomy cohorts are shown in Table 1. Percentagewise, more subjects were current smokers or obese, consumed alcohol, or had hypertension or DM in the non-appendectomy vs. appendectomy cohort; however, the absolute differences were small. The proportion of the subjects diagnosed with GI cancers did not differ between the cohorts.

**Table 1:** Demographic characteristics of subjects with and without appendectomy.

Variables	Appendectomy		*p* value
	Yes (n = 158,101)	No (n = 474,303)	
	No. (%)	No. (%)	
**Age group**			1
< 20 yr	585 (0.37)	1,755 (0.37)	
20–39 yr	65,267 (41.28)	195,801 (41.28)	
40–59 yr	67,524 (42.71)	202,572 (42.71)	
60–79 yr	23,478 (14.85)	70,434 (14.85)	
≥ 80 yr	1,247 (0.79)	3,741 (0.79)	
Sex (male)	90,143 (57.02)	270,429 (57.02)	1
**Smoking**			< .0001
Never-smoker	94,792 (59.96)	279,412 (58.91)	
Ex-smoker	23,385 (14.79)	64,274 (13.55)	
Current-smoker	39,924 (25.25)	130,617 (27.54)	
Alcohol	98,347 (62.21)	296,646 (62.54)	0.016
Obesity (BMI ≥ 25 kg/m^2^)	49,801 (31.50)	150,776 (31.79)	0.032
Hypertension	32,869 (20.79)	104,398 (22.01)	< .0001
DM	9,924 (6.28)	35,507 (7.49)	< .0001
Dyslipidaemia	22,628 (14.31)	67,441 (14.22)	0.357
**GI cancers**	1,403 (0.89)	4,135 (0.87)	0.564
Oesophageal cancer	70 (0.04)	185 (0.04)	0.366
Gastric cancer	686 (0.43)	2,117 (0.45)	0.519
Small bowel cancer	25 (0.02)	68 (0.01)	0.675
Colorectal cancer	699 (0.44)	2,000 (0.42)	0.280

*yr* years, *BMI* body mass index, *DM* diabetes mellitus, *GI* gastrointestinal.

### Incidence of GI cancers in the overall population

The incidence of all GI cancers did not differ significantly in the non-appendectomy and appendectomy cohorts (1.529 and 1.557 per 1,000 person-years [PY], respectively) (Table 2). Risk analyses identified age, sex, obesity, current smoking, alcohol consumption, hypertension, DM, and dyslipidaemia as risk factors for GI cancers in both crude and multiple regression models.

**Table 2:** Crude and adjusted hazard ratios and 95% confidence intervals for the incidence of gastrointestinal cancers in the overall population.

			GI cancers						
		No	Event	Duration	IR	Crude model	*p *value	aHR (95% CI)	*p *value
Age	1 yr					1.071 (1.069–1.073)	< .0001	1.074 (1.072–1.076)	< .0001
Sex	Male	360,572	3,686	2,057,945	1.791	1	< .0001	1	< .0001
	Female	271,832	1852	1,547,498	1.197	0.669 (0.632–0.707)		0.550 (0.517–0.586)	
Obesity	No	431,827	3,520	2,475,933	1.422	1	< .0001	1	0.004
	Yes	200,577	2018	1,129,509	1.787	1.259 (1.191–1.329)		1.086 (1.027–1.149)	
Current-smoker	No	461,863	4,120	2,621,761	1.571	1	0.005	1	< .0001
	Yes	170,541	1,418	983,682	1.442	0.916 (0.863–0.973)		1.154 (1.080–1.234)	
Alcohol	No	237,411	1941	1,159,896	1.673	1	< .0001	1	< .0001
	Yes	394,993	3,597	2,445,546	1.471	0.851 (0.805–0.901)		1.150 (1.082–1.223)	
Hypertension	No	495,137	3,168	2,838,344	1.116	1	< .0001	1	0.015
	Yes	137,267	2,370	767,099	3.090	2.773 (2.629-.924)		1.077 (1.014–1.143)	
DM	No	586,973	4,624	3,361,149	1.376	1	< .0001	1	< .0001
	Yes	45,431	914	244,293	3.741	2.733 (2.546–2.934)		1.272 (1.180–1.370)	
Dyslipidaemia	No	542,335	4,379	3,126,954	1.400	1	< .0001	1	0.042
	Yes	90,069	1,159	478,488	2.422	1.742 (1.633–1.859)		0.932 (0.871–0.997)	
Appendectomy	No	474,303	4,135	2,704,515	1.529	1	0.550	1	0.281
	Yes	158,101	1,403	900,928	1.557	1.019 (0.959–1.082)		1.034 (0.973–1.098)	

*aHR* adjusted hazard ratio, *CI* confidence interval, *GI* gastrointestinal, *IR* incidence rate (1000 person-years), *yr* year, *DM* diabetes mellitus.

**Figure 2:**
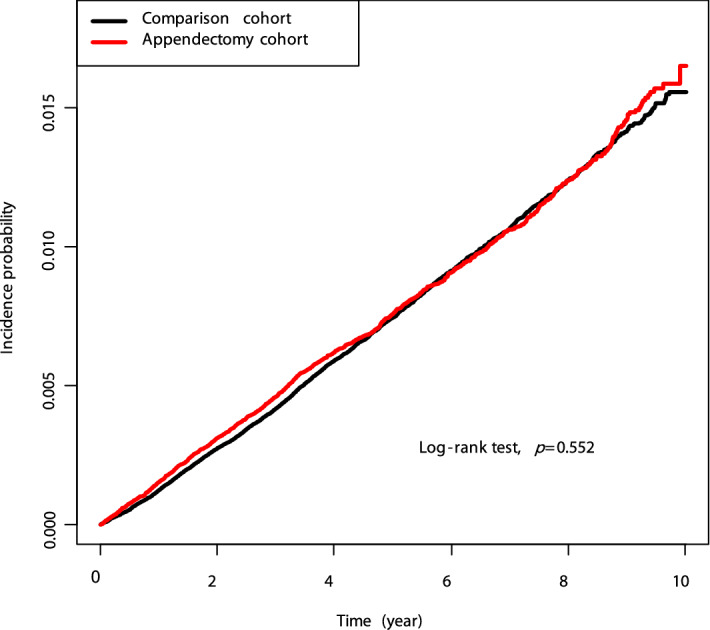
Kaplan–Meier curves of incidence probability for gastrointestinal cancers according to appendectomy in the overall population.

Risk analysis was also performed for each type of GI cancers. In the non-appendectomy and appendectomy cohorts, the incidence ratios per 1,000 PY were 0.068 and 0.077 for oesophageal cancer, 0.781 and 0.760 for gastric cancer, 0.025 and 0.028 for small bowel cancer, and 0.738 and 0.774 for colorectal cancer, respectively. There was no significant association between appendectomy and any type of GI cancers in analyses adjusted for age, sex, obesity, current smoking, alcohol consumption, hypertension, DM, and dyslipidaemia (Table 3). Kaplan–Meier curves showed no significant difference in the cumulative proportional incidence of GI cancers, either as a whole (Fig. 2) or per type, between the non-appendectomy and appendectomy cohorts.

**Table 3:** Adjusted hazard ratios and 95% confidence intervals for the incidence of each type of gastrointestinal cancers in the overall population.

		Oesophageal cancer			Gastric cancer			Small bowel cancer			Colorectal cancer		
		Event	IR	aHR (95% CI)	Event	IR	aHR (95% CI)	Event	IR	aHR (95% CI)	Event	IR	aHR (95% CI)
Age	1 yr			1.112 (1.101–1.124)			1.076 (1.073–1.079)			1.054 (1.037–1.071)			1.069 (1.065–1.072)
Sex	Male	228	0.110	1	2005	0.972	1	52	0.025	1 (Ref.)	1623	0.787	1
	Female	27	0.017	0.179 (0.117- 0.274)	798	0.515	0.444 (0.405–0.486)	41	0.026	0.881 (0.554–1.399)	1,076	0.694	0.695 (0.637–0.758)
Obesity	No	184	0.074	1	1797	0.724	1	64	0.026	1 (Ref.)	1675	0.675	1
	Yes	71	0.063	0.830 (0.626–1.100)	1,006	0.888	1.072 (0.991–1.16)	29	0.026	0.836 (0.533–1.309)	1,024	0.904	1.138 (1.051–1.233)
Current-smoker	No	130	0.049	1	2038	0.776	1	74	0.028	1 (Ref.)	2099	0.799	1
	Yes	125	0.127	2.863 (2.193–3.737)	765	0.776	1.154 (1.053–1.264)	19	0.019	0.906 (0.518–1.585)	600	0.609	1.053 (0.952–1.165)
Alcohol	No	60	0.052	1	921	0.792	1	33	0.028	1 (Ref.)	1,033	0.889	1
	Yes	195	0.079	1.649 (1.212–2.244)	1882	0.768	1.207 (1.107–1.316)	60	0.024	1.248 (0.782–1.993)	1666	0.680	1.04 (0.953–1.133)
Hypertension	No	136	0.048	1	1638	0.576	1	54	0.019	1 (Ref.)	1516	0.533	1
	Yes	119	0.154	1.085 (0.829–1.419)	1,165	1.513	1.02 (0.938–1.109)	39	0.050	1.315 (0.82–2.109)	1,183	1.536	1.131 (1.038–1.233)
DM	No	212	0.063	1	2,336	0.694	1	79	0.023	1 (Ref.)	2,245	0.667	1
	Yes	43	0.174	1.132 (0.807–1.587)	467	1.903	1.303 (1.175–1.446)	14	0.057	1.307 (0.719–2.376)	454	1.849	1.294 (1.165–1.439)
Dyslipidaemia	No	207	0.066	1	2,270	0.725	1	74	0.024	1 (Ref.)	2066	0.659	1
	Yes	48	0.100	0.898 (0.648–1.244)	533	1.110	0.848 (0.769–0.936)	19	0.039	0.942 (0.553–1.605)	633	1.319	1.048 (0.954–1.151)
Appendectomy	No	185	0.068	1	2,117	0.781	1	68	0.025	1 (Ref.)	2000	0.738	1
	Yes	70	0.077	1.174 (0.892–1.546)	686	0.760	0.987(0.906–1.076)	25	0.028	1.112 (0.703–1.759)	699	0.774	1.065 (0.977–1.161)

*aHR* adjusted hazard ratio, *CI* confidence interval, *GI* gastrointestinal, *IR* incidence rate (1000 person-years), *yr* year, *DM* diabetes mellitus.

### Subgroup analyses for the incidence of GI cancers

We also analysed patients stratified by three risk factors for GI cancers: age (< 60 years vs. ≥ 60 years), sex (male vs. female), and DM (non-DM vs. DM). The incidence of all GI cancers was significantly higher in the appendectomy cohort (5.342 per 1,000 PY) than in the non-appendectomy (4.910 per 1,000 PY) cohort, showing aHR, 1.102 (95% CI of 1.011–1.201, *p* = 0.028) in elderly (≥ 60 years) subgroup. It was also significantly higher in the appendectomy cohort (1.346 per 1,000 PY) than in the non-appendectomy cohort (1.147 per 1,000 PY), showing aHR of 1.180 (95% CI of 1.066–1.306, *p* = 0.001) in female subgroup (Table 4).

**Table 4:** Crude and adjusted hazard ratios and 95% confidence intervals for the incidence of gastrointestinal cancers in the subgroups.

Subgroup	Appendectomy	GI cancers							
		No	Event	Duration	IR	Crude model	*p *value	aHR (95% CI)	*p* value
Age < 60 yr	No	400,128	2,179	2,306,181	0.945	1	0.347	1	0.503
	Yes	133,376	697	768,762	0.907	0.960 (0.881–1.045)		0.971 (0.892–1.058)	
Age ≥ 60 yr	No	74,175	1956	398,333	4.910	1	0.055	1	0.028
	Yes	24,725	706	132,166	5.342	1.088 (0.998–1.186)		1.102 (1.011–1.201)	
Male	No	270,429	2,803	1,543,388	1.816	1	0.147	1	0.386
	Yes	90,143	883	514,557	1.716	0.946 (0.877–1.020)		0.967 (0.897–1.043)	
Female	No	203,874	1,332	1,161,127	1.147	1	0.002	1	0.001
	Yes	67,958	520	386,371	1.346	1.173 (1.06–1.298)		1.180 (1.066–1.306)	
Non-DM	No	438,796	3,427	2,513,769	1.363	1	0.284	1	0.377
	Yes	148,177	1,197	847,380	1.413	1.037 (0.971–1.107)		1.03 (0.965–1.100)	
DM	No	35,507	708	190,746	3.712	1	0.651	1	0.454
	Yes	9,924	206	53,548	3.847	1.037 (0.888–1.210)		1.061 (0.909–1.239)	

*aHR* adjusted hazard ratio, *CI* confidence interval, *GI* gastrointestinal, *IR* incidence rate (1000 person-years), *yr* years, *DM* diabetes mellitus.

Table 5 presents the subgroup analyses for each type of GI cancers. Appendectomy did not significantly correlate with gastric or small bowel cancer in any of the subgroups. However, it was significantly associated with oesophageal cancer in the female subgroup (aHR, 2.742; 95% CI, 1.289–5.835; *p* = 0.009) and with colorectal cancer in the elderly (aHR, 1.145; 95% CI, 1.012–1.295; *p* = 0.032) and the female (aHR, 1.243; 95% CI, 1.09–1.418; *p* = 0.001) subgroups.

**Table 5:** Crude and adjusted hazard ratios and 95% confidence intervals for the incidence of each type of gastrointestinal cancers in the subgroups.

Subgroup	Appendectomy	Oesophageal cancer			Gastric cancer			Small bowel cancer			Colorectal cancer		
		Event	IR	aHR (95% CI)	Event	IR	aHR (95% CI)	Event	IR	aHR (95% CI)	Event	IR	aHR (95% CI)
Age < 60 yr	No	74	0.032	1	1,114	0.482	1	44	0.019	1	1,072	0.464	1
	Yes	23	0.030	0.978 (0.612–1.562)	345	0.448	0.943 (0.836–1.064)	12	0.016	0.824 (0.435–1.561)	353	0.459	0.997 (0.884–1.124)
Age ≥ 60 yr	No	111	0.275	1	1,003	2.503	1	24	0.059	1	928	2.314	1
	Yes	47	0.351	1.288 (0.915–1.812)	341	2.563	1.033 (0.914–1.169)	13	0.097	1.621 (0.825–3.186)	346	2.599	1.145 (1.012–1.295)
Male	No	171	0.110	1	1526	0.987	1	39	0.025	1	1,237	0.799	1
	Yes	57	0.110	1.042 (0.772–1.406)	479	0.929	0.962 (0.868–1.066)	13	0.025	1.006 (0.537–1.887)	386	0.748	0.959 (0.855–1.075)
Female	No	14	0.012	1	591	0.508	1	29	0.025	1	763	0.656	1
	Yes	13	0.034	2.742 (1.289–5.835)	207	0.535	1.056 (0.901–1.237)	12	0.031	1.239 (0.632–2.428)	313	0.809	1.243 (1.09–1.418)
Non-DM	No	152	0.060	1	1753	0.696	1	59	0.023	1	1647	0.654	1
	Yes	60	0.071	1.181 (0.876–1.593)	583	0.687	0.980 (0.892–1.076)	20	0.024	0.997 (0.601–1.657)	598	0.704	1.070 (0.975–1.175)
DM	No	33	0.171	1	364	1.899	1	9	0.047	1	353	1.842	1
	Yes	10	0.185	1.134 (0.558–2.303)	103	1.915	1.035 (0.831–1.288)	5	0.093	1.986 (0.665–5.934)	101	1.877	1.041 (0.834–1.299)

*aHR* adjusted hazard ratios, *CI* confidence intervals, *GI* gastrointestinal, *IR* incidence rate (1000 person-years), *yr* years, *DM* diabetes mellitus.

We further stratified the subjects by sex and age into 4 subgroups (males under 60 years, females under 60 years, males ≥ 60 years and females ≥ 60 years) to minimize possible confounding effect which might arose from disproportion of age distribution within the female subgroup and disproportion of sex distribution within the elderly subgroups. Appendectomy did not increase the risk of overall GI cancers in the 3 subgroups except the elderly female subgroup (aHR 1.229, 95% CI of 1.066–1.418). As regards to each type of GI cancers, the appendectomy cohort showed higher aHR for oesophageal cancer (aHR, 4.432; 95% CI of 1.576–12.462) and colorectal cancer (aHR 1.349; 95% CI of 1.119–1.626) compared to the non-appendectomy cohort within the elderly female subgroup. However, appendectomy did not increase risk of small bowel cancer and gastric cancer within the elderly female subgroup (Supplementary Table S1 online).

## Discussion

This large-scale population-based cohort study showed no significant association between appendectomy and GI cancer in the overall population. However, there was a significant association between appendectomy and GI cancers within elderly patients ≥ 60 years and within female patients. In analyses adjusted for BMI, smoking status, alcohol consumption, hypertension, DM, and dyslipidaemia, the appendectomized female subgroup was susceptible to oesophageal cancer and colorectal cancer, whereas the appendectomized elderly subgroup was susceptible to colorectal cancer.

The appendix houses a diverse colonic microbiome and, as a gut-associated lymphoid tissue, is part of the immune system. Therefore, its removal might decrease microbial diversity and hamper host immune function. Appendectomized subjects might be susceptible to dysbiosis and unable to mount a microbial response to counteract or adapt to the dysbiosis owing to loss of the microbiome. By altering metabolite levels and impairing host immune responses, persistent dysbiosis can promote the development of various diseases such as inflammatory bowel disease, celiac disease, cardiovascular disease, DM, rheumatoid arthritis, osteoarthritis, and even neurologic disorders via the brain–gut axis^20–27^. A significant association between appendectomy and these diseases has been observed in some large cohort studies^28–34^.

Dysbiosis has also been linked to lung, breast, and GI cancers^5–8,12–14^. Because interactions between the microbiome and the host are more direct in the GI tract than in other organs, we can postulate that appendectomy critically affects the pathogenesis of malignancy by interrupting the microbial ecosystem in the GI tract. However, only a few studies have examined the risk of GI cancers after appendectomy, and the results are conflicting^15,17,35^.

Although appendectomy did not increase the risk of GI cancers in our entire study cohort, it did increase risk in the elderly and female subgroups. Along with intestinal physiologic and nutritional changes, intestinal microbe diversity decreases in the elderly^36,37^. Furthermore, age-related impairment of immune function could enable the evolution of the bacterial strains responsible for dysbiosis and elderly-specific infectious conditions ^36–38^. Biofilms are most abundant in the appendix, followed in order by the cecum and ascending colon^3,4^. Taken together, the microbial ecosystem would not be resilient after appendectomy owing to its depletion; moreover, the underlying diminished microbial diversity caused by aging could accelerate and perpetuate dysbiosis.

Differences in microbial composition between men and women have been reported by the Human Microbiome Project consortium, the Belgian Flemish Gut Flora Project, and the Dutch LifeLines-DEEP study; all found greater α-diversity in women^39,40^. Although the incidence of GI cancers is lower in women than in men, microbial adaptation or resilience and consequent eubiosis might be more challenging in appendectomized women than in women with an intact appendix in post-infectious conditions.

This study has some limitations. First, incidental appendectomy was not differentiated from appendectomy due to appendicitis; therefore, we could not assess the impact of incidental appendectomy in the non-inflammatory context. Second, irritable bowel disease and pre-existing adenomas were not taken into account in the risk analysis. Considering the fact that irritable bowel disease might be suppressed by appendectomy and colonic adenomas are premalignant lesions, they are potential hidden confounding factors^28,29^. Third, the follow-up period in our study was shorter than the conventional follow-up period of 10 years, which is based on the time frame (7–10 years on average) of the adenoma to carcinoma transition^15,41,42^. However, in the large cohort study by Wu et al.^15^, the overall colorectal cancer incidence was highest during the first 1.5–3.5 years after appendectomy; therefore, a median follow-up duration of 5.6 (3.78–7.59) years is acceptable. Further, we applied one year of lag time for wash-out to exclude patients with pre-existing but undetected cancers at the index date.

In conclusion, this large-scale population-based study showed that appendectomy did not increase the risk of GI cancers in the overall population. However, it identified elderly patients and female patients as at-risk subpopulations owing to their higher rates of GI cancers after appendectomy.

## Supplementary information

Supplementary Information.

## Data source

This study used NHIS data (NHIS-2019-1-089) provided by the NHIS in South Korea.
