# Publisher Correction: A link between appendectomy and gastrointestinal cancers: a large-scale population-based cohort study in Korea

**DOI:** 10.1038/s41598-021-85952-6

**Published:** 2021-03-17

**Authors:** Youn Young Park, Kil‑yong Lee, Seong Taek Oh, Sang Hyun Park, Kyung Do Han, Jaeim Lee

**Affiliations:** 1 Division of Coloproctology, Department of Surgery, College of Medicine, Uijeongbu St. Mary’s Hospital, The Catholic University of Korea, Cheonbo‑ro 271, Uijeongbu‑si, Gyeonggi‑do 11765 Republic of Korea; 2 Department of Medical Statistics, College of Medicine, Catholic University of Korea, Seoul, Republic of Korea; 3 Department of Statistics and Actuarial Science, Soongsil University, Seoul, Republic of Korea

Correction to: *Scientific Reports* https://doi.org/10.1038/s41598-020-72770-5, published online 24 September 2020

The original version of this Article contained a typographical error in the spelling of the author Youn Young Park, which was incorrectly given as You Young Park.

Secondly, the Abstract contained an error.

Appendectomy did not significantly increase the incidence of GI cancers in the overall population (1.529 and 1557 per 1000 person-years in the non-appendectomy and appendectomy cohorts, respectively)

now reads:

Appendectomy did not significantly increase the incidence of GI cancers in the overall population (1.529 and 1.557 per 1000 person-years in the non-appendectomy and appendectomy cohorts, respectively).

Furthermore, Figure 2 contained an error.

The incorrect Figure 2 appears below as Figure 1.

**Figure 1:**
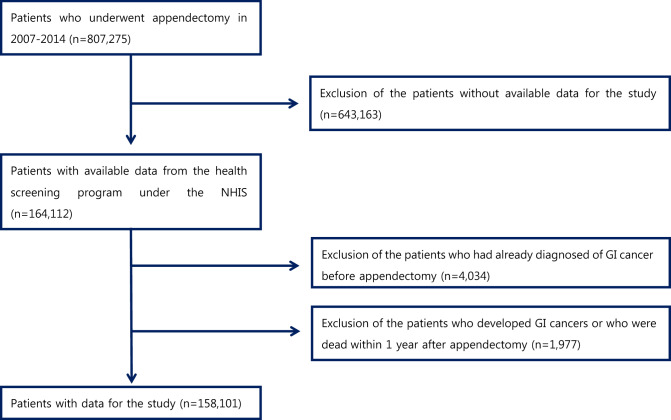
Kaplan–Meier curves of incidence probability for gastrointestinal cancers according to appendectomy in the overall population.

Lastly, the Data Availability statement was incorrectly given as Funding information.

These errors have now been corrected in the PDF and HTML versions of the Article.

