# Supplementary material for: A link between appendectomy and gastrointestinal cancers: a large-scale population-based cohort study in Korea
Source: Sci Rep. 2020 Sep 24;10:15670. doi: 10.1038/s41598-020-72770-5 (PMC7518248; doi:10.1038/s41598-020-72770-5)
Supplement: Supplementary file 1 — Supplementary Information. [file 41598_2020_72770_MOESM1_ESM.docx]

Supplementary Table S1. Adjusted hazard ratios and 95% confidence intervals for the incidence of gastrointestinal cancers in the subgroups stratified by age and sex.

|  | | | aHR (95% CI) | | | | |
| --- | --- | --- | --- | --- | --- | --- | --- |
|  |  |  | GI cancers | Oesophageal cancer | Gastric cancer | Small bowel cancer | Colorectal cancer |
| Age <60yr & male | Age | 1yr | 1.119 (1.113-1.125) | 1.232 (1.185-1.281) | 1.121 (1.112-1.130) | 1.084 (1.041-1.129) | 1.109 (1.100-1.119) |
|  | Obesity | No | 1 | 1 | 1 | 1 | 1 |
|  |  | Yes | 1.101 (1.005-1.208) | 0.979 (0.626-1.531) | 1.053 (0.930-1.192) | 0.816 (0.397-1.676) | 1.140 (0.994-1.308) |
|  | Current-smoker | No | 1 | 1 | 1 | 1 | 1 |
|  |  | Yes | 1.184 (1.081-1.296) | 2.786 (1.783-4.355) | 1.227 (1.086-1.385) | 0.558 (0.264-1.180) | 1.104 (0.964-1.266) |
|  | Alcohol | No | 1 | 1 | 1 | 1 | 1 |
|  |  | Yes | 1.173 (1.042-1.322) | 3.635 (1.460-9.055) | 1.159 (0.989-1.358) | 0.696 (0.328-1.479) | 1.109 (0.929-1.325) |
|  | Hypertension | No | 1 | 1 | 1 | 1 | 1 |
|  |  | Yes | 1.133 (1.024-1.254) | 1.467 (0.933-2.304) | 1.019 (0.888-1.170) | 0.724 (0.309-1.696) | 1.288 (1.109-1.496) |
|  | DM | No | 1 | 1 | 1 | 1 | 1 |
|  |  | Yes | 1.378 (1.209-1.569) | 1.101 (0.609-1.990) | 1.516 (1.278-1.799) | 2.577 (1.058-6.277) | 1.297 (1.064-1.582) |
|  | Dyslipidaemia | No | 1 | 1 | 1 | 1 | 1 |
|  |  | Yes | 0.921 (0.813-1.043) | 1.109 (0.645-1.904) | 0.893 (0.754-1.058) | 1.072 (0.422-2.723) | 0.960 (0.798-1.154) |
|  | Appendectomy | No | 1 | 1 | 1 | 1 | 1 |
|  |  | Yes | 0.899 (0.808-0.999) | 0.911 (0.547-1.519) | 0.898 (0.779-1.035) | 0.512 (0.198-1.325) | 0.905 (0.772-1.060) |
| Age <60yr & female | Age | 1yr | 1.074 (1.066-1.083) | 1.066 (1.003-1.133) | 1.074 (1.061-1.086) | 1.050 (1.001-1.102) | 1.076 (1.066-1.087) |
|  | Obesity | No | 1 | 1 | 1 | 1 | 1 |
|  |  | Yes | 1.118 (0.964-1.297) | 0.296 (0.037-2.350) | 1.131 (0.895-1.430) | 0.654 (0.230-1.862) | 1.170 (0.969-1.414) |
|  | Current-smoker | No | 1 | 1 | 1 | 1 | 1 |
|  |  | Yes | 1.422 (1.049-1.929) | 6.363 (1.353-29.925) | 1.333 (0.817-2.175) | 1.140 (0.152-8.576) | 1.308 (0.867-1.974) |
|  | Alcohol | No | 1 | 1 | 1 | 1 | 1 |
|  |  | Yes | 1.155 (1.007-1.326) | 0.614 (0.162-2.333) | 1.281 (1.031-1.592) | 1.864 (0.759-4.579) | 1.071 (0.897-1.278) |
|  | Hypertension | No | 1 | 1 | 1 | 1 | 1 |
|  |  | Yes | 0.982 (0.827-1.167) | 0.484 (0.059-4.006) | 0.986 (0.749-1.297) | 2.660 (0.985-7.187) | 0.935 (0.749-1.167) |
|  | DM | No | 1 | 1 | 1 | 1 | 1 |
|  |  | Yes | 1.096 (0.826-1.454) | ㅡ* | 1.069 (0.671-1.702) | 2.606 (0.702-9.677) | 1.170 (0.827-1.655) |
|  | Dyslipidaemia | No | 1 | 1 | 1 | 1 | 1 |
|  |  | Yes | 0.924 (0.766-1.115) | 1.486 (0.301-7.335) | 0.713 (0.517-0.984) | 0.914 (0.280-2.983) | 1.064 (0.845-1.340) |
|  | Appendectomy | No | 1 | 1 | 1 | 1 | 1 |
|  |  | Yes | 1.133 (0.980-1.309) | 1.477 (0.445-4.906) | 1.081 (0.859-1.360) | 1.410 (0.575-3.459) | 1.150 (0.955-1.385) |
| Age ≥60yr & male | Age | 1yr | 1.038 (1.030-1.046) | 1.049 (1.022-1.077) | 1.034 (1.023-1.045) | 1.003 (0.925-1.089) | 1.039 (1.027-1.051) |
|  | Obesity | No | 1 | 1 | 1 | 1 | 1 |
|  |  | Yes | 1.063 (0.959-1.179) | 0.805 (0.542-1.196) | 1.051 (0.92-1.212) | 1.064 (0.388-2.918) | 1.136 (0.974-1.326) |
|  | Current-smoker | No | 1 | 1 | 1 | 1 | 1 |
|  |  | Yes | 1.197 (1.075-1.333) | 2.720 (1.945-3.804) | 1.102 (0.948-1.280) | 2.049 (0.781-5.379) | 1.083 (0.915-1.282) |
|  | Alcohol | No | 1 | 1 | 1 | 1 | 1 |
|  |  | Yes | 1.110 (1.002-1.230) | 1.334 (0.922-1.930) | 1.141 (0.990-1.314) | 1.163 (0.397-3.406) | 0.992 (0.850-1.157) |
|  | Hypertension | No | 1 | 1 | 1 | 1 | 1 |
|  |  | Yes | 1.142 (1.034-1.262) | 0.908 (0.646-1.277) | 1.057 (0.923-1.211) | 1.830 (0.662-5.056) | 1.226 (1.053-1.429) |
|  | DM | No | 1 | 1 | 1 | 1 | 1 |
|  |  | Yes | 1.145 (1.019-1.287) | 1.036 (0.671-1.599) | 1.076 (0.912-1.268) | 0.478 (0.108-2.122) | 1.274 (1.074-1.511) |
|  | Dyslipidaemia | No | 1 | 1 | 1 | 1 | 1 |
|  |  | Yes | 0.842 (0.748-0.949) | 0.688 (0.435-1.088) | 0.782 (0.661-0.925) | 1.308 (0.451-3.790) | 0.965 (0.812-1.147) |
|  | Appendectomy | No | 1 | 1 | 1 | 1 | 1 |
|  |  | Yes | 1.037 (0.931-1.156) | 1.103 (0.761-1.599) | 1.031 (0.889-1.197) | 2.422 (0.953-6.142) | 1.016 (0.861-1.199) |
| Age ≥60yr & female | Age | 1yr | 1.047 (1.037-1.058) | 1.058 (0.981-1.141) | 1.052 (1.037-1.068) | 1.054 (0.985-1.129) | 1.042 (1.028-1.056) |
|  | Obesity | No | 1 | 1 | 1 | 1 | 1 |
|  |  | Yes | 1.074 (0.938-1.229) | 0.433 (0.120-1.566) | 1.033 (0.846-1.262) | 0.814 (0.303-2.184) | 1.141 (0.954-1.364) |
|  | Current-smoker | No | 1 | 1 | 1 | 1 | 1 |
|  |  | Yes | 1.267 (0.844-1.902) | 6.016 (1.334-27.124) | 1.124 (0.600-2.017) | 2.472 (0.327-18.686) | 1.153 (0.650-2.047) |
|  | Alcohol | No | 1 | 1 | 1 | 1 | 1 |
|  |  | Yes | 1.057 (0.914-1.223) | 2.651 (0.934-7.526) | 1.196 (0.969-1.476) | 1.627 (0.622-4.259) | 0.933 (0.765-1.139) |
|  | Hypertension | No | 1 | 1 | 1 | 1 | 1 |
|  |  | Yes | 0.976 (0.850-1.119) | 1.259 (0.426-3.715) | 0.989 (0.808-1.211) | 1.222 (0.473-3.156) | 1.003 (0.835-1.205) |
|  | DM | No | 1 | 1 | 1 | 1 | 1 |
|  |  | Yes | 1.285 (1.092-1.513) | 1.313 (0.359-4.797) | 1.341 (1.056-1.704) | 0.624 (0.141-2.758) | 1.266 (1.020-1.571) |
|  | Dyslipidaemia | No | 1 | 1 | 1 | 1 | 1 |
|  |  | Yes | 1.008 (0.877-1.158) | 1.026 (0.340-3.099) | 0.861 (0.697-1.062) | 0.546 (0.177-1.679) | 1.170 (0.975-1.403) |
|  | Appendectomy | No | 1 | 1 | 1 | 1 | 1 |
|  |  | Yes | 1.229 (1.066-1.418) | 4.432 (1.576-12.462) | 1.035 (0.832-1.288) | 1.048 (0.377-2.912) | 1.349 (1.119-1.626) |
| Abbreviations: aHR, adjusted hazard ratio; CI, confidence interval; GI, gastrointestinal; yr. year; DM, diabetes mellitus  *, not available due to zero event | | | | | | | |
